# Assessing Elementary School Students’ Manipulative Skill Competency in China

**DOI:** 10.3390/ijerph18063150

**Published:** 2021-03-18

**Authors:** Jun Chen, Xiaozan Wang, Zhanjia Zhang, Weiyun Chen

**Affiliations:** 1 College of Physical Education and Health, East China Normal University, Shanghai 200241, China; michaelsara2011@163.com (J.C.); xzwang@tyxx.ecnu.edu.cn (X.W.); 2 Key Laboratory of Adolescent Health Assessment and Exercise Intervention of Ministry of Education, East China Normal University, Shanghai 200241, China; 3 School of Kinesiology, University of Michigan, 830 N University Avenue, Ann Arbor, MI 48109, USA; zzj@umich.edu

**Keywords:** manipulative skill competency, validation, skill assessment

## Abstract

This study aimed to test the validity and reliability of the Physical Education (PE) Metric Assessment Rubrics for assessing 4th-grade students’ manipulative skill competency and examine how well they demonstrated manipulative skill competency. Participants were 4th-grade students at six elementary schools in China. A total of 535 4th-grade students were assessed their soccer skills and 819 4th-grade students were assessed their basketball skills using the PE-Metric Assessment Rubrics. The results found that the soccer and basketball skill assessments had a high degree of internal consistency. And the results showed that the soccer and basketball skill assessment rubrics had a good factor structure. The students’ mean score in soccer and basketball skills assessment was lower than the Overall Competent Level. Further, the *t*-test indicated that soccer and basketball skill assessments had a significant difference in the mean score of Overall Competent Level between the boys and the girls. The PE-Metric Assessment Rubrics were valid and reliable assessment tools for assessing the manipulative skill competency in soccer and basketball skills among 4th-grade students in China. This study suggested that Chinese elementary school students need to improve basic manipulative skill competency in soccer and basketball skills.

## 1. Introduction

Motor skill competency is essential to developing an active lifestyle for youth [1]. Motor skills consist of locomotor skills, manipulative skills, and non-manipulative skills. Among them, manipulative skills can be defined as the ability to use hand or other body parts, and objects to control an object (e.g., strike, dribble, kick, throw) [2,3,4]. Previous studies have indicated that development of motor skill competency during childhood is related to regular physical activity in school-aged children and adolescents [1,5,6,7,8,9]. Wrotniak et al. [5] found that children’s motor skill proficiency assessed with Bruininks-Oseretsky Test of Motor proficiency was significantly associated with moderate-to-vigorous physical activity. Okely et al. [6] found that competency in fundamental movement skill was positively associated with engagement in organized physical activity in adolescents. Furthermore, Barnett et al. [3] found that children’s motor skill competency, especially manipulative skill competency predicted adolescents’ participation in organized and moderate-to-vigorous physical activity. Due to the global trend of physical inactivity among school-aged children and adolescents [10,11,12,13], it is very important to develop manipulative skill competency in childhood.

According to the National Standard 1 for Physical Education (PE), demonstrating motor skill competency and using motor skills in dynamic and complex environment is one of the major desired performance outcomes for elementary school students to be able to achieve [14]. There are many motor skill assessment tools designed to evaluate students’ motor skill competency, such as The Bruininks-Oseretsky Test of Motor Proficiency (BOTMP) [15], and Test of Gross Motor Development (TGMD) [16]. However, these assessment tools use either product-oriented criteria to assess the outcome of the skill execution or process-oriented criteria to assess the critical components of each motor skill [15,16]. The PE Metrics are a valid and reliable assessment rubrics based on National PE Standard 1 to measure students’ manipulative skill competency, using both process- and product-oriented criteria [17].

Recently, researchers have used the PE Metrics Assessment Rubrics to assess elementary school students’ motor skills competency in locomotor and manipulative skills [18,19,20,21,22,23]. For example, Chen et al. used the PE Metric Assessment Rubrics to assess 1223–1588 of K-1 students’ motor skill competency in running skill, hand dribbling skill, weight transferring, and underhand catching skill [22], and 565 of 4th-grade students’ manipulative skill competency in soccer skills, overhand throwing skill, striking skill with a paddle, and basketball skills [21]. In another study by Chen et al. [18], 2723 of fourth- and fifth-grade students were assessed in terms of their soccer skills, 3420 of fourth- and fifth-grade students were assessed in terms of their baseball overhand throwing, and 2709 of fourth- and fifth-grade students were assessed in terms of their tennis striking skill with the PE Metrics Assessment Rubrics. Similarly, Zhang et al. [23] used the PE Metric assessment rubrics to assess 215 underserved Hispanic fourth- and fifth- grade students’ motor skill competency in basketball, striking, and overhand throwing. Furthermore, Chen et al. [19] indicated that the PE Metrics Assessment Rubrics are feasible tools for PE teachers to assess levels of students’ motor skill competency during a regular PE lesson. However, there is a lack of studies that use PE Metrics Assessment Rubrics to evaluate elementary school students’ manipulative skill competency in China.

To the best of our knowledge, no study has used the PE Metrics Assessment Rubrics to assess 4th-grade students’ demonstration of manipulative skill competency in China. Thus, the purpose of this study was twofold: (a) testing validity and reliability of the PE Metric Assessment Rubrics for assessing 4th-grade students’ manipulative skill competency in China, and (b) examining how well 4th-grade students demonstrated manipulative skill competency, as well as gender difference of 4th-grade students’ manipulative skill competency assessed with selected the PE Metric Assessment Rubrics.

## 2. Methods

### 2.1. Participants and Settings

In this study, a total of 535 4th-grade students (307 boys vs. 228 girls) aged 9–11 years old voluntarily completed the PE Metrics soccer dribbling, passing, and receiving skills test. A total of 819 4th-grade students (450 boys vs.369 girls) aged 9–11 years old voluntarily completed the PE Metrics basketball dribbling, passing, and receiving skills test. The PE teachers’ ages varied from 27–50 years old with a range of 7–30 years of teaching experience. Each school principal granted the permission for their school to participate in this study. The University Institutional Review Board (HUM00149529) approved the study protocols. Written informed consent was obtained from all students’ parent/guardian.

### 2.2. Data Collection

#### 2.2.1. PE Metrics Assessment Rubrics

Two manipulative skills selected from the PE metrics and assessed in this study were soccer dribbling, passing, and receiving skills, and basketball dribbling, passing, and receiving skills. Each assessment rubric has its specific essential dimensions, performance indicators (assessment criteria for each level of the rating scale) on 0–4 rating scales, and the number of trial for testing [17]. Each rubric contains four levels of performance that range from proficient (level 4) to lacking in competence (level 1) [24].

For the soccer dribbling, passing, and receiving skills assessment, one trial was allowed for the test. Criteria for Competence (level 3) in Dribbling is: “dribble with control while moving at a slow, consistent jog”, for Passing is: “sends a receivable lead pass to a partner so it can be received outside the passing lane without a break in the receiver’s stride on at least 3 passes”, and for Receiving is: “moves forward and outside the passing lane to meet the ball while receiving at least 3 receivable passes” [17] (p. 120). A total score of 9 indicated an Overall Competent Level. The Maximum score is 12.

For the basketball dribbling, passing, and receiving skills assessment, one trial was allowed for the test. Criteria for Competence (level 3) for Dribbling is: “dribbles with control while moving at a slow, consistent jog, ” for Passing is: “sends a catchable lead pass to a partner so it can be caught outside the passing lane without a break in the receiver’s stride on at least 3 passes, ” and for Receiving is: “moves forward and outside the passing lane to meet the ball while receiving at least 3 receivable passes” [17] (p. 98). A total score of 9 indicated an Overall Competent Level. The maximum score is 12.

#### 2.2.2. Collection of Students’ Performances in Soccer and Basketball Skills

Prior to the fall semester of 2018, the six elementary school PE teachers were trained in learning the two manipulative skill assessment rubrics, and assessment protocols for soccer and basketball skills, how to organize students to perform soccer skills and basketball skills assessment tasks. For example, during administration of each test, first, the PE teacher demonstrated and explained how to take the test based on the assessment protocols. Next, the PE teacher organized the students into each pair and gave each pair two practice trials to ensure all students understand the assessment tasks. Last, the PE teacher organized each pair to take the test which was video-recorded using the digital camera [17]. A total of 535 students in the fall semester of 2018 and spring semester of 2019 completed the soccer dribbling, passing, and receiving skills assessment. A total of 819 students in the fall semester of 2018 and spring semester of 2019 completed the basketball dribbling, passing, and receiving skills assessment.

#### 2.2.3. Evaluation of Students’ Performance in Soccer and Basketball Skills

After two pairs of research assistants received 4-h training in learning and practicing of coding four students’ performance in soccer and basketball skills using the PE Metric Assessment Rubrics, the inter-rater reliability (IR) reached ≥80% between the two coders for coding each of the four students’ soccer and basketball skills. The IR of the coded soccer or basketball skills assessment was examined by checking each evaluator’s coding results using the formula: % IR = (number of agreement ÷ (numbers of agreement + numbers of disagreement)) × 100 [25].

Then, two research assistants used the PE Metrics Assessment Rubrics to assess 535 4th-grade students’ soccer skill competency through coding each video-recorded students’ performance in soccer dribbling, passing, and receiving skills on a 0–4 rating scale. Moreover, the other two research assistants used the PE Metrics Assessment Rubrics to assess 819 4th-grade students’ basketball skill competency through watching each video-recorded students’ performance in basketball dribbling, passing, and receiving skills on the 0–4 rating scale. The two research assistants in each pair watched the recorded video together, but each research assistant independently coded each video-recorded students’ performance in both soccer and basketball skills. Each research assistant was required to strictly use the PE Metrics Assessment Rubrics [17] to conduct the two manipulative skill assessments with all participating students.

### 2.3. Data Analysis

SPSS (Version 26.0; IBM Cooperation, Armonk, NY, USA) was used to conduct all analysis of the data. First, to determine the reliability of the soccer and basketball skill assessments, Cronbach’s alpha coefficients were used to analyze the coding data. Moreover, to examine the relationships between the three essential dimensions (dribbling, passing, receiving) and the total score of the soccer and basketball skill assessments, Pearson correlation coefficients was conducted. To analyze the validity of the soccer and basketball skill assessments, exploratory factor analysis (EFA) was conducted. In EFA analysis, construct validity were tested by The Kaiser–Meyer–Olkin (KMO) Measure of Sampling Adequacy and The Bartlett’s Test of Sphericity [26]. Value of The KMO measure of sampling adequacy more than or equal 0.70 is considered adequate, and values of The Bartlett’s test of sphericity less than 0.05 are conduct an EFA [27]. In this study, the one factor of dribbling, passing, receiving were used to determine the pattern of structure measurement of the soccer skills, basketball skills with eigenvalue, respectively [28]. In addition, Comrey et al. [29] indicated that a sample size of 300 is good to conduct EFA.

Then, descriptive statistics and percentages were computed to determine the levels and proportions of the students’ demonstration of competency in soccer and basketball skill assessments. An independent *t*-test was conducted to examine the mean score difference in soccer and basketball skill assessments between the boys and the girls.

## 3. Results

### 3.1. Reliability and Validity of the PE Metric Assessment Rubrics

#### 3.1.1. Soccer Skill Assessments

Table 1 presents the descriptive statistics, Pearson’s correlations, and Cronbach alpha correlation coefficients of the two manipulative skill assessments. The total score of the soccer skill assessments was represented by the sum of the dribbling, passing, receiving. As seen in Table 1, all items showed a normal distribution with skewness and kurtosis values less than 2.00. Results of the bivariate correlation coefficients between the three essential dimensions indicated that association between dribbling and passing, dribbling and receiving, passing and receiving were strongly linked to each other at *p* < 0.01 level. Each essential dimension was strongly correlated with the total score at *p* < 0.01 level. The Cronbach alpha coefficients for the total score was 0.879 and the alpha correlation coefficients of the three essential dimensions ranged from 0.792 to 0.852. The results indicated that the soccer skill assessments had a high degree of internal consistency [27].

Table 2 presents the EFA of the two manipulative skill assessments. For soccer skill assessments, The Bartlett’s test of sphericity was significant (x^2^_(3)_ = 1000.976, *p* < 0.001) showed that there was patterned relationship between the dribbling, passing, and receiving. The KMO measure of sampling adequacy was 0.69 indicating the data were sufficient for principal components analysis. A total of one factor had eigenvalues greater than 1.00, the factor loadings of the soccer dribbling, passing, and receiving skills ranged from 0.828 to 0.931. As seen in Table 2, eigenvalue for the one factor extracted was 2.417, explaining a cumulative variance of 80.562. The results indicated that the soccer skill assessment rubrics has a good factor structure.

#### 3.1.2. Basketball Skill Assessments

The total score of the basketball skill assessments was represented by the sum of the three essential dimensions. As seen in Table 1, the results showed a normal distribution with skewness and kurtosis values less than 2.00. Pearson correlation coefficients showed dribbling, passing, receiving were correlated to each other, ranging from r = 0.630–0.857. Each essential dimension also was strongly correlated with the total score, ranging from r = 0.819–0.933. Cronbach alpha coefficients for total score, dribbling, passing, receiving were 0.922, 0.833, 0.828, 0.815. The results indicated that the basketball skill assessments had a high degree of internal consistency [27].

As presented in Table 2, for basketball skill assessments, the results of Bartlett’s test of sphericity showed that there was patterned relationship between the dribbling, passing, and receiving (x^2^_(3)_ = 1857.716, *p* < 0.001). And the results of KMO measure of sampling adequacy indicated that the data were sufficient for principal components analysis. A total of one factor had eigenvalues greater than 1.00, the factor loadings for basketball dribbling, passing, and receiving skills were 0.943, 0.930, 0.918. Moreover, this three-item structure was found to explain a cumulative variance of 86.516. The results indicated that the basketball skill assessment rubrics had a good factor structure.

### 3.2. Descriptive Statistics and Gender Differences of the Manipulative Skill Competency

#### 3.2.1. Soccer Skill Competency

Table 3 presents the descriptive statistics of the two manipulative skill assessments separated by gender. For the soccer dribbling, passing, and receiving skills assessment, a total score of 9/12 indicated the Overall Competent Level. Of 535 students who completed the soccer skills test, 176 (36.9%) students reached the Overall Competent Level or above (>9/12). As seen in Table 3, 36.2% of 111 boys and 28.5% of 65 girls demonstrated the Overall Competent Level or above in soccer skills. The boys’ mean score (M = 7.279, SD = 3.2819) was lower than the Overall Competent level. Similarly, the girls’ mean score (M = 6.721, SD = 2.9586) was lower than the Overall Competent level. The independent sample *t*-test showed a significant difference in the mean total score of soccer skills between the boys and girls (*t* = −2.024, *p* = 0.043).

#### 3.2.2. Basketball Skill Competency

For the basketball dribbling, passing, and receiving skills assessment, a total score of 9/12 indicated the Overall Competent Level. As seen in Table 3, 819 students who completed the basketball skills test, 355 (43.3%) students reached the Overall Competent Level or above (>9/12). Moreover, 45.8% of 206 boys and 40.4% of 149 girls demonstrated the Overall Competent Level or above in basketball skills. The boys’ and girls’ mean score (M = 7.744, SD = 2.9127; M = 6.745, SD = 3.4686) was lower than the Overall Competent level. The results of *t*-test showed a significant difference in the mean total score between the boys and girls (*t* = −4.405, *p* = 0.000).

## 4. Discussion

The central purpose of this study were to test the reliability and validity of the PE Metric Assessment Rubrics for assessing elementary school students’ manipulative skill competency in China, and to examine how well 4th-grade students demonstrated manipulative skill competency and gender difference of 4th-grade students’ manipulative skill competency. Examining the Cronbach’s alpha reliability coefficients of PE-Metric Assessment Rubrics indicated that both soccer and basketball assessments had satisfactory internal consistency reliability. Consistent with the results in the present study, previous studies have shown that soccer and basketball skill assessments had satisfactory internal consistency reliability [20,21]. For example, Chen et al. [20] showed that alpha reliability coefficient of the soccer skills assessments was 0.92. Similarly, another study by Chen et al. [21] showed that alpha reliability coefficient of soccer skill assessments was 0.90, and that of basketball skill assessments was 0.92.

Examining the relationships between the three essential dimensions and total score of the soccer skill assessments indicated that dribbling, passing, receiving and total score are highly related to each other. Similarly, examining the relationships between the three essential dimensions and total score of the basketball skill assessments indicated that dribbling, passing, receiving and the total score are highly associated with each other. The EFA showed that soccer and basketball skill assessments had a sound construct validity. Consistent with previous studies [30,31,32,33], our study confirmed that the PE-Metric Assessment Rubrics are reliable and valid tools for assessing 4th-grade students’ manipulative skill competency in soccer and basketball skills in China.

It is important to note that in our study the students’ mean scores in soccer skills and basketball skills were lower than the Overall Competent Level. It appears that 4th graders in the study had not mastered manipulative skills related to soccer and basketball. Consistent with the results of our study, previous studies [18,31] have also shown that American students’ manipulative skill competency was lower than the Overall Competent Level. For example, Zhu et al. [31] found that 581 fifth-grade students’ mean score in soccer skill assessment was lower than the Overall Competent Level (M = 6.17, SD = 2.7), 238 fifth-grade students’ mean score in basketball skill assessment was also lower than the Overall Competent Level (M = 7.10, SD = 2.99). Similarly, Hushman et al. [18] found that 47 fifth-grade students’ mean score in basketball skill assessment was slightly lower than the Overall Competent Level (M = 8.60, SD = 2.28), 42 fifth-grade students’ mean score in soccer skill assessment was also lower than the Overall Competent Level (M = 7.20, SD = 2.22). In contrast, Chen et al., found that 2723 fourth- and fifth-grade students’ mean score in soccer skill assessments was higher than the Overall Competent Level (M = 9.48, SD = 1.90) [20], 565 of 4th-grade students’ mean score in basketball skill assessments was higher than the Overall Competent Level (M = 10.11, SD = 1.66) [21]. Studies have showed that children’s manipulative skill competency was related to physical activity in adolescent and adulthood [7,9,34,35]. In addition, dribbling, passing, receiving skills are basic manipulative skills commonly used in soccer or basketball games. Therefore, our study recommend that PE teacher should use quality PE curriculum to help elementary school students improve basic soccer and basketball skills as well as use the PE-Metric Assessment rubrics to assess students’ manipulative skill competency. Moreover, PE teacher should design specific soccer or basketball games for students to have more opportunities for using the dribbling, passing, receiving skills to develop their manipulative skill competency.

Further, our study found that the boys significantly outperformed the girls in soccer and basketball skill assessments. The proportions of the boys demonstrating the Overall Competent Level or above were higher than those of the girls in soccer and basketball skills. In line with the results of our study, previous studies [18,19,21,36] showed that boys demonstrated better levels of manipulative skills than girls. For example, Chen et al. [21] found that boys were more competent in performing soccer skills, basketball skills, overhand throwing skill, and striking skill with a paddle than girls. Likewise, Chen et al. [19] showed that boys’ mean scores in soccer skills, overhand throwing skill, and striking skill were significantly higher than for the girls’. Similarly, Hushman et al. [18] found that boys were more competent in soccer skills, basketball skills, and overhand throwing skill than girls. Consistent with the results above, Hardy et al. [36] found that Australian boys performed better than girls on striking a stationary ball, catching skill, kicking skill, overhand throwing skill, but girls performed better than boys on locomotor skills (run, gallop, hop, horizontal jump). In addition, Okely et al. [6] reported that girls who were more competent in performing manipulative skills continued to engage in organized physical activity during adolescence. however, girls with less competence in manipulative skills were less likely to participate in organized sports during adolescence. This is because manipulative skills such as overhand throwing, catching, dribbling, and passing skills are foundational skills applied in playing a variety of sports and other organized physical activities.

The study has several implications in existing literature. First, this study provides an empirical evidence that the PE-Metric Assessment Rubrics are reliable and valid assessment tools that can be used to assess elementary school students’ manipulative skill competency in China. In addition, the results of this study show that students’ mean score in two manipulative skills are lower than the Overall Competent Level. This study suggests that it is important to develop elementary school students’ manipulative skill competency in soccer and basketball skills.

This study has two limitations: (a) this study only focused on testing the elementary school students’ soccer and basketball skills with selected PE-Metric Assessment Rubrics. The future study could test the validity and reliability of the PE-Metric Assessment Rubrics for assessing students’ other manipulative skill competency (e.g., overhand throwing skill, tennis striking skill) in China; and (b) it should be noted that the sample size of this study, which is not enough to represent the mean score of elementary school students’ manipulative skill competency in China. In future studies, it is necessary to expand the sample size to test elementary school students’ manipulative skill competency in more regions of China.

## 5. Conclusions

This study established that the PE Metric Assessment Rubrics are valid and reliable tools for assessing elementary school students’ manipulative skill competency in China. The 4th-grade students demonstrated lower than the Overall Competent Level in soccer and basketball skill assessments. This study indicated that the boys significantly outperformed the girls on soccer and basketball skill assessments. This study suggests that Chinese elementary school students need to improve basic manipulative skill competency in soccer and basketball skills.

## Figures and Tables

**Table 1:** Descriptive statistics, Pearson’s correlations, and alpha coefficients of the manipulative skill assessments.

PE-Metric Assessment Rubric	Skewness	Kurtosis	Pearson Correlation				α
			Dribbling	Passing	Receiving	Total Score	
Soccer							
Dribbling	−0.408	−0.361	1				0.852
Passing	−0.745	−0.246	0.631 **	1			0.798
Receiving	−0.470	−0.707	0.630 **	0.857 **	1		0.792
Total score	−0.796	−0.059	0.819 **	0.929 **	0.933 **	1	0.879
Basketball							
Dribbling	−0.245	−0.865	1				0.833
Passing	−0.324	−0.856	0.765 **	1			0.828
Receiving	−0.270	−1.045	0.799 **	0.829 **	1		0.815
Total score	−0.288	−0.962	0.917 **	0.929 **	0.944 **	1	0.922

Note: ** represents *p* < 0.01.

**Table 2:** Exploratory factor analyses of the manipulative skill assessments: factor loading from the structure matrix.

PE-Metric Assessment Rubric	Factor 1	Eigenvalue	% of Variance	% of Cumulative
Soccer				
Dribbling	0.931	2.417	80.562	80.562
Passing	0.930			
Receiving	0.828			
Basketball				
Dribbling	0.943	2.595	86.516	86.516
Passing	0.930			
Receiving	0.918			

**Table 3:** Descriptive statistics of the manipulative skill assessments for total sample and by sex.

PE-Metric Assessment Rubric	Total		Boys		Girls	
	M (SD)	# of OCL (% of OCL)	M (SD)	# of OCL (% of OCL)	M (SD)	# of OCL (% of OCL)
Soccer						
Dribbling	1.995 (1.0234)	139 (26.0%)	2.088 (1.0704)	97 (31.6%)	1.871 (0.9443)	42 (18.4%)
Passing	2.619 (1.2103)	290 (54.2%)	2.717 (1.2444)	182 (59.3%)	2.487 (1.1524)	108 (47.4%)
Receiving	2.434 (1.2545)	240 (44.9%)	2.490 (1.2897)	147 (47.9%)	2.357 (1.2041)	93 (40.8%)
Total score	7.041 (3.1574)	176 (32.9%)	7.279 * (3.2819)	111 (36.2%)	6.721 (2.9586)	65 (28.5%)
Basketball						
Dribbling	2.463 (1.1185)	405 (49.5%)	2.609 (1.0475)	241 (53.6%)	2.286 (1.1768)	164 (44.4%)
Passing	2.436 (1.1340)	420 (51.3%)	2.598 (1.0481)	247 (54.9%)	2.238 (1.2029)	173 (46.9%)
Receiving	2.395 (1.1998)	421 (51.4%)	2.538 (1.1001)	248 (55.1%)	2.221 (1.2914)	173 (46.9%)
Total score	7.294 (3.2120)	355 (43.3%)	7.744 ** (2.9127)	206 (45.8%)	6.745 (3.4686)	149 (40.4%)

Note: * Boy means higher than girl means at the alpha 0.05 level; ** Boy means significantly higher than girl means at the alpha 0.01 level; OCL = Overall Competent Level; M = Mean; SD = Standard deviation.

## Data Availability

The data presented in this study are available on request from the corresponding author.
